# Youth Who Control HIV on Antiretroviral Therapy Display Unique Plasma Biomarkers and Cellular Transcriptome Profiles Including DNA Repair and RNA Processing

**DOI:** 10.3390/cells14040285

**Published:** 2025-02-15

**Authors:** Samiksha A. Borkar, Li Yin, Guglielmo M. Venturi, Jerry Shen, Kai-Fen Chang, Bernard M. Fischer, Upasana Nepal, Isaac D. Raplee, John W. Sleasman, Maureen M. Goodenow

**Affiliations:** 1 Molecular HIV and Host Interactions Section, National Institute of Allergy and Infectious Diseases, National Institutes of Health, 50 South Drive, Bethesda, MD 20894, USA; li.yin@nih.gov (L.Y.); jerry.shen@nih.gov (J.S.); kai-fen.chang@nih.gov (K.-F.C.); nepal.upasana@nih.gov (U.N.); isaac.raplee@nih.gov (I.D.R.); maureen.goodenow@nih.gov (M.M.G.); 2 Division of Allergy and Immunology, Department of Pediatrics, Duke University School of Medicine, Durham, NC 27710, USA; guglielmo.venturi@duke.edu (G.M.V.); bernie.fischer@duke.edu (B.M.F.); john.sleasman@duke.edu (J.W.S.)

**Keywords:** youth with HIV, viral suppression, bioprofiles, biomarkers, transcriptome, inflammation, comorbidities, DNA repair, cellular homeostasis

## Abstract

Combination antiretroviral therapy (ART) suppresses detectible HIV-1 replication, but latent reservoirs and persistent immune activation contribute to residual viral-associated morbidities and potential viral reactivation. youth with HIV (YWH) virally suppressed on ART early in infection before CD4 T cell decline with fewer comorbidities compared to adults represent a critical population for identifying markers associated with viral control and predictors of viral breakthrough. This study employed a multi-omics approach to evaluate plasma biomarkers and cellular gene expression profiles in 52 participants, including 27 YWH on ART for 144 weeks and 25 youth with no infection (NI) (ages 18–24). Among the 27 YWH, 19 were virally suppressed (VS; <50 RNA copies/mL), while eight were non-suppressed (VNS; >50 RNA copies/mL). VS YWH displayed unique bioprofiles distinct from either VNS or NI. Early viral suppression mitigates inflammatory pathways and normalizes key biomarkers associated with HIV-related comorbidities. Genes upregulated in pathways linked to cellular homeostasis such as DNA repair, RNA processing, and transcription regulation may diminish viral breakthrough and maintain sustained HIV control on ART. Candidate markers and putative molecular mechanisms were identified, offering potential therapeutic targets to limit viral persistence, enhance HIV treatment strategies, and pave the way for improved clinical outcomes.

## 1. Introduction

Combination antiretroviral therapy (ART) prevents HIV-1 disease progression by optimal suppression of viral replication below the levels of detection in plasma by routine clinical testing. However, adverse viral effects persist, primarily due to latent viral reservoirs in resting memory CD4 T cells, monocytes, macrophages, and anatomical reservoirs, such as the brain and the gastrointestinal tract. Viral reservoirs are poised for potential reactivation under suitable host cell conditions [1,2,3]. Moreover, immune activation attributed to residual HIV replication [4] or spontaneous HIV RNA and protein expression [5] contributes to viral associated morbidities. Identifying biomarkers of sustained viral suppression could enable more precise treatment approaches to minimize HIV-associated disease progression.

Most of the studies examining bioprofiles of viral suppression have focused on chronically infected adults with HIV who have been living with viral infection for many years and have age-related comorbidities, and/or substance use that could modulate the ART-mediated viral suppression bioprofiles [6]. Few studies have focused on youth with HIV (YWH), age 13 to 24, who comprise over 19% of all new HIV infections in the United States [7]. YWH who have acquired infection through sexual transmission are early in disease progression and have fewer comorbidities compared to their adult counterparts. The bioprofiles of individuals with sustained viral suppression (VS) (HIV-1 RNA <50 copies/mL) who initiated ART before CD4 T cell decline or the development of HIV-associated comorbidities could help identify targets or markers associated with viral control and predictors of viral breakthrough.

Monitoring plasma biomarkers of immune activation, such as cytokines and chemokines expressed by T cells and macrophages, markers of intestinal integrity, and vascular damage can provide valuable insights into disease pathogenesis [8,9,10,11,12]. In addition, gene expression profiles of host responses associated with the control of viral replication holds the potential to enhance the discovery of host–virus interactions and identify therapeutic targets to limit viral persistence and improve HIV treatment outcomes. Recent studies on plasma biomarkers in people with HIV who initiated ART after immunosuppression reported increased IL-8 levels, a marker of chronic inflammation compared to healthy controls [13]. Transcriptome analyses of individuals on ART highlighted host gene expression patterns in memory CD4 T cells that promote HIV silencing and cell survival compared to healthy controls [14]. Pollara et al. showed that genes regulating inflammatory processes and controlling viral replication are perturbed during ART in older adults in peripheral blood mononuclear cells [15].

There is a significant knowledge gap in research focusing on the bioprofiles of youth with HIV. Understanding the unique viral suppression bioprofiles of this age group after initial ART will provide new insight into the continuum of disease progression across the lifespan. This study used a multi-omics approach to define the candidate biomarkers and genes that profiles viral suppression in plasma and peripheral blood cells (PBC) of youth with HIV on ART. The hypothesis is that plasma biomarker and cellular gene expression profiles in youth with HIV (YWH) with viral suppression (VS) are distinct from YWH with non-suppressed viral replication (VNS) or youth with no infection (NI). This study found that individual bioprofiles highlight key factors influencing sustained viral suppression. Persistent inflammation, indicated by elevated biomarkers across multiple immune pathways, may impede effective viral suppression on ART in non-suppressed youth, while early viral suppression may help normalize inflammatory pathways and key biomarkers linked to HIV-related comorbidities. Genes upregulated in pathways linked to cellular homeostasis, such as DNA repair, RNA processing, and transcription regulation, may diminish viral breakthrough and maintain sustained HIV control on ART.

## 2. Materials and Methods

### 2.1. Demographics and Clinical Characteristics Among the Study Participants

The study cohort consisted of 129 YWH, aged 18–24 years with behaviorally acquired HIV infection enrolled between 2010 and 2013 at 22 urban centers across the United States and Puerto Rico (ClinicalTrials.gov; https://clinicaltrials.gov/, ClinicalTrails-Identifier No NCT00491556 and NCT00683579) under the Adolescent Medicine Trials Network (ATN) for HIV/AIDS Intervention protocols 061 and 071/101. The enrollment procedure and main outcome results for this three-year longitudinal study were previously published [8,16,17,18,19,20,21,22,23,24]. Treatment-naïve participants early in disease with CD4 T cell counts >350 cells/μL initiated combination ART regimens at entry. The regimens included a protease inhibitor (ritonavir-boosted atazanavir) plus two nucleotide reverse transcriptase inhibitors (emtricitabine and tenofovir) or a non-nucleoside reverse transcriptase inhibitor (efavirenz). The participants were examined for clinical status, viral load, CD4 T cell counts and antiretroviral medications at entry and weeks 24, 48, 96 and 144 (end-of-study) time points. Peripheral blood samples collected at the end of study were used to assess the plasma biomarkers and peripheral blood cell transcriptome.

The current analysis focuses on a nested study of a sub-group of 27 YWH selected based on their viral load status at the end of study and absence of substance use validated by toxicology and self-report. The groups included 19 YWH with sustained viral suppression (VS) (<50 HIV-1 RNA copies/mL) for at least one year prior to and at the end of study, and 8 YWH with non-suppressed viral replication (VNS) (>50 HIV-1 RNA copies/mL) on the same therapy. Among the VNS group, 2 participants had persistent viral replication throughout the study, while the other 6 VNS participants experienced intermittent increases in viral load, suggestive of virological breakthrough. Additionally, this study included a single blood sample from a reference group of 25 youth with no HIV infection (NI) balanced with YWH for gender, race and no substance use, acute illnesses, recent vaccinations, or pregnancy [8,16,19].

The majority of the study population consisted of African American participants (82%) and males (75%) (Table 1). Median plasma HIV-1 RNA (viral load) for the VS or VNS outcome groups was 22,607 copies/mL or 16,504 copies/mL at entry. By the end of study, all participants in the VS group achieved viral suppression, while the VNS group had a median viral load of 6706 copies/mL.

**Figure 1:**
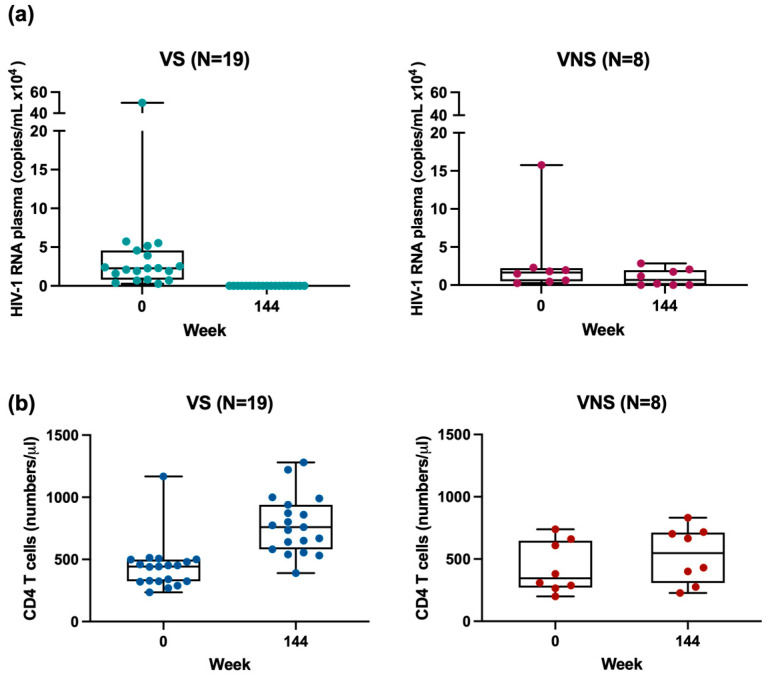
Plasma HIV-1 RNA (viral load) and CD4 T cell number for YWH. (**a**) Viral load distribution at entry and end of study for VS and VNS YWH; (**b**) CD4 T cell numbers at entry and end of study for VS and VNS YWH.

The absolute number and percentages of lymphocyte subpopulations were measured using flow cytometry, as previously described [20]. Based on CD45 gating to identify total lymphocytes, percentages and the absolute number of total CD4 T cells, CD8 T cells and CD19 B cells, were enumerated with further gating applied to identify naïve and effector memory CD4 T cells within the total CD4 population. The percentages of lymphocyte subpopulations of total CD4 T cells, naïve and effector memory CD4 T cells, total CD8 T cells and total CD19 B cells at the end of study were comparable between VS and VNS groups of YWH. (Table S1). The median CD4 T cell counts at entry and nadir CD4 T cell counts were comparable between the VS and VNS groups. The end of study CD4 T cell counts were similar between YWH and NI, with the median counts within normal ranges [25]. The VS CD4 counts increased over time on treatment, while VNS remained at pretreatment levels over time. Regardless of their clinical outcomes at the end of study, both VS and VNS YWH maintained stable CD4 counts, with no decline even at baseline before ART was initiated (Figure 1).

### 2.2. Multiplex Assays for Plasma Biomarkers

To define the inflammatory bioprofiles, 23 plasma biomarkers were measured using Meso Scale Diagnostics, Gaithersburg, MD, USA, according to the manufacturer’s protocol [26,27]. The measured biomarkers were associated with macrophage activation (CRP, GM-CSF, sCD14 and sCD163), T cell activation and differentiation (sCD27, IFNγ, and IL-2RA), IFNγ-inducible chemokines (CXCL9 and CXCL10), inflammatory chemokines (CCL2, CCL4, CCL5 and IL-8), inflammatory cytokines (TNFα, IL-1β and IL-6), anti-inflammatory cytokines (IL-10), intestinal barrier dysfunction (iFABP and LBP), neutrophil function (MPO), vascular inflammatory biomarkers (sVCAM-1 and sICAM-1) and cellular migration (MMP-2), broadly associated with immune activation [8].

### 2.3. Unsupervised and Supervised Machine Learning and Feature Selection

Unsupervised principal component analysis (PCA), scaled by calculating the z-score of the plasma biomarker data (mean concentration in pg/mL), was performed. The first three principal components were visualized, and the two components which best separated the groups were presented. Data points were colored by groups and ellipses for each group were generated with a 95% confidence interval for each group. R package Plotly version 4.10.3 [https://plot.ly, accessed on 10 August 2024] and ggplot2 version 3.5.1 [https://ggplot2.tidyverse.org, accessed on 12 August 2024] were used for graphical display and interpretation [28,29,30].

For supervised machine learning, the random forest (RF) classification algorithm was employed for feature selection to identify the most predictive biomarkers that differentiate VS, VNS, and NI. Measurements below the lower limit of detection (LOD) were included without imputation or transformation, maintaining data integrity for optimal feature selection and classification. Learning curves which define the effectiveness of the RF model were generated using Python package scikit-learn version 1.6 [31]. The RF model was trained on a 70–30% training–test split with the number of decision trees equal to 100. The performance of the model was assessed using Area Under the Curve of the Receiver Operator Characteristic (AUC-ROC) using scikit-learn and the matplotlib package version 3.10 [31,32] (Figure S1). Most predictive biomarkers (fifteen top ranking) were plotted with a beeswarm plot using Shapley Additive Explanation (SHAP) [33] package version 0.46.0 in Python 3.9.12, Scotts Valley, CA, USA [34]. The SHAP program assigns an importance value to each biomarker, indicating its relative contribution and directional impact on the final classification model.

### 2.4. RNA Isolation, Microarray Hybridization and Scanning

Total intracellular RNA was isolated from blood collected in PAXgene Blood RNA tubes with PAXgene Blood RNA Kit (PreAnalytiX, Hombrechtikon, Switzerland). Followed by globin-mRNA being depleted, 100 ng of RNA was amplified using GeneChip 3′ IVT Express Kit (Affymetrix Genechip, Santa Clara, CA, USA) and hybridized to the Gene Chip Human Genome U133 Plus 2.0 Array with 54,675 probes representing 20,174 genes at the Interdisciplinary Center for Biotechnology Research (ICBR) at the University of Florida. Raw CEL format images were produced using the Affymetrix GeneChip Operating Software version 1.4, Santa Clara, CA, USA. The quality of each array was assessed by manually inspecting mean values, variances, and paired scatter plots. All arrays met the quality check criteria. Raw probe signal values were corrected for background noise, quantile normalized, and summarized using the Robust Multi-Array Averaging (RMA) method, implemented in R with Bioconductor package 3.20 [35]. All expression values were log_2_ transformed for further analysis. Log_2_-transformed expression values were used to visualize RNA expression levels of genes encoding highly significant plasma biomarkers with violin plots using GraphPad Prism 10.0.3 (GraphPad Software, San Diego, CA, USA).

### 2.5. Differential Gene Expression Analysis

The Significance Analysis of Microarrays (SAM) version 3.0 was used to identify the genes with statistically significant changes in expression level between the study groups. YWH VS group (n = 19) was compared with VNS (n = 8) as a reference group, and the VS and VNS YWH groups were each compared with the NI group (n = 25) as a reference. The SAM package uses 1000 permutations of repeated measurements to estimate the false discovery rate (FDR), which indicates the proportion of genes identified as significant purely by chance [36]. Fold change (FC) for each gene was calculated by determining the average expression value for each study group, followed by computing the anti-log2 of the difference between the study group and the reference group. Genes with an absolute FC ≥ 1.3 and FDR < 0.05 were designated as differentially expressed genes (DEGs) [37,38]. To study the relationship between the DEGs, a Venn diagram was composed using R package VennDiagram version 1.7.3, which enables the generation of highly customizable Venn diagrams [39]. A heatmap of 13 shared genes was plotted using R package pheatmap version 1.0.12 [https://CRAN.R-project.org/package=pheatmap, accessed on 20 August 2024].

### 2.6. Functional Enrichment and Network Analysis of Differentially Expressed Genes

To infer the functions of the DEGs, functional enrichment analysis was performed using the Database for Annotation, Visualization and Integrated Discovery (DAVID) (v2023q4, MD, USA), https://david.ncifcrf.gov, accessed on 2 September 2024), which integrates pathway information from the Kyoto Encyclopedia of Genes and Genomes (KEGG) and Gene-Ontology (GO term) database. DAVID provides a comprehensive set of functional annotation tools to infer the biological meaning behind large lists of genes [40]. The cutoff value for pathway screening and significant functionality was set to *p* < 0.001. The networks of the most prominent pathways and DEGs were visualized by R package ClusterProfiler version 4.14.4 with a gene-concept network plot [41].

### 2.7. Candidate DEGs Within Perturbed Pathways That Profiles Viral Suppression

Candidate genes perturbed in the key pathways or across multiple pathways were visualized as network plots using Cytoscape version 3.10.1, a software program for visualizing molecular networks and gene expression profiles [42]. The HIV-1 human interaction database and GeneCard were used as the resources for the gene function information [43,44].

### 2.8. Statistical Analysis

One-way ANOVA was used to compare across the study groups (age, ART duration, CD4 count, percentages of lymphocyte subpopulations, top biomarkers and RNA expression levels (log_2_-transformed values) of genes encoding highly significant plasma biomarkers). T-test was used to compare between the two groups. Chi-square test was utilized to measure the impact of gender and race on the HIV status, viral load status and ART regimen in YWH. GraphPad Prism 10.0.3 (GraphPad Software, San Diego, CA, USA) was employed for all the statistical analyses. Statistical significance was defined by *p* value < 0.05. The viral loads and CD4 counts at baseline and the end of study (week 144) were plotted using GraphPad Prism.

## 3. Results

### 3.1. Plasma Profiles Reveal Immune Activation and Intestinal Dysfunction in VS Compared with VNS or NI

**Figure 2:**
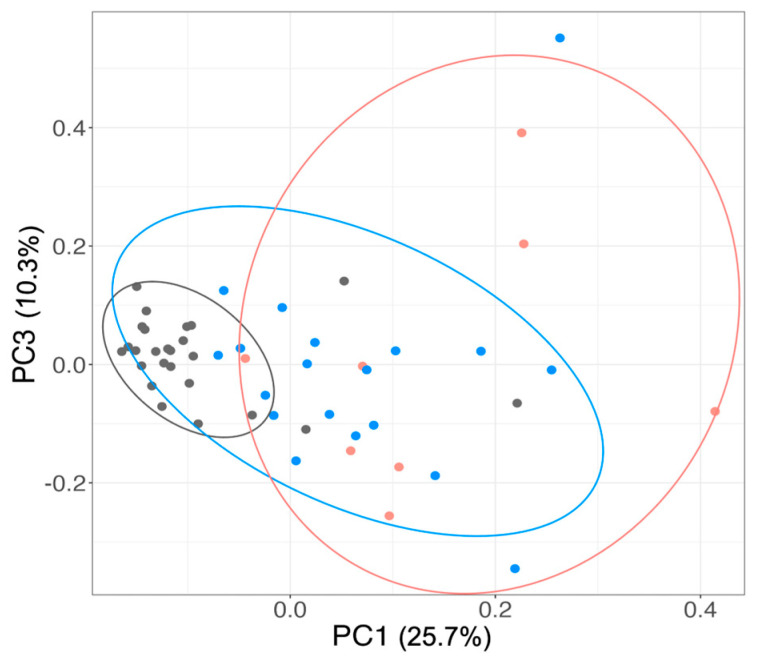
Unsupervised PCA based on 23 plasma biomarker data. To visualize the variance among the study groups, biomarker data scaled by calculating the z-score was applied to the PCA. The ellipses were generated with 95% confidence intervals for each group. Symbols: dot, participants ellipses, participant clusters. Grey dot: NI; Blue dot: VS; Pink dot: VNS. Grey ellipses: 22 NI with 3 VS and 1 VNS; Blue ellipses: 14 VS with 3 NI and 4 VNS; Pink ellipses: 3 VNS and 2 VS.

To identify biomarkers associated with viral suppression, multivariate analyses, including PCA, random forest classification with SHAP interpretation, and one-way ANOVA, were performed to distinguish among the VS, VNS and NI groups. PCA applied to 23 plasma biomarkers showed that the first three principal components collectively explained almost half (49.2%) of the original variance, with PC1, PC2 and PC3 collectively accounting for 25.7%, 13.22% and 10.3% of variance, respectively. PC1 and PC3 separated the study participants into three clusters (Figure 2). Most participants from the NI group (22/25; 88%) clustered together, while most YWH (23/27; 85%) formed a separate cluster, indicating a clear distinction between the groups based on viral infection. However, the PCA analysis of the plasma biomarker was less effective in distinguishing VS or VNS status among YWH. None of the outcome clusters by PCA were entirely homogenous, suggesting variability within the groups.

**Figure 3:**
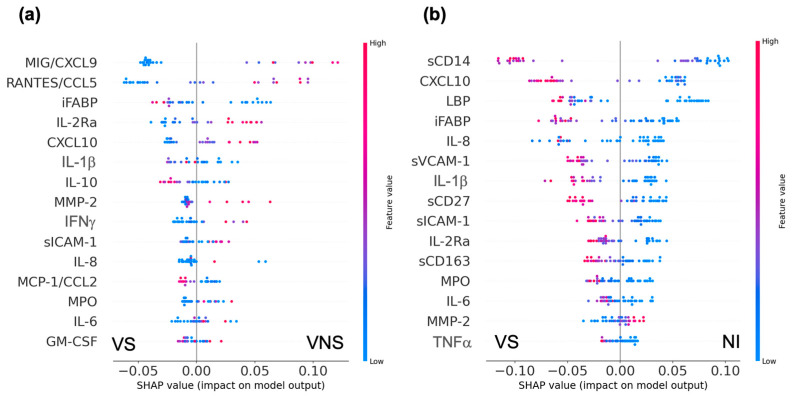
Random forest classification model. Beeswarm plot of RF model showing the top fifteen biomarkers that are more predictive of VS compared with VNS (**a**) or with NI (**b**). Biomarkers are ranked by their SHAP value in descending order of their relative contribution to the classification model. Each point on the plot represents the biomarker measurement from a single participant. Larger SHAP values correspond to a larger contribution of a biomarker in classification of a participant. The classification model for VS compared with VNS showed an AUC-ROC of 1.0, while VS or VNS compared with NI each showed an AUC-ROC of 0.9.

Using the random forest classification model, the top predictive biomarkers distinguishing between VS and VNS were the interferon-stimulated chemokines CXCL9 and CCL5, the intestinal barrier dysfunction marker iFABP, the T cell activation marker IL-2RA and the interferon-inducible chemokine CXCL10 (Figure 3a). A VS profile differed from a VNS profile by lower concentrations of CXCL9 and CCL5. In contrast, the VS profile differed from NI by higher concentrations of macrophage activation marker sCD14, interferon-inducible chemokine CXCL10, and intestinal barrier dysfunction markers LBP and iFABP, vascular inflammation marker sVCAM-1, inflammatory cytokine IL-1β, and T cell activation marker (sCD27), but a lower concentration of inflammatory chemokine IL-8 (Figure 3b). Between VNS and NI, higher levels of CXCL9, CXCL10, LBP, and sCD14 were associated with a higher likelihood of a VNS profile (Figure S2). The random forest analysis highlighted that the plasma biomarker profile of the VS group was distinct from both the VNS and NI groups.

The significance of the top biomarkers distinguishing VS from VNS or NI was further assessed to discern the group differences in plasma concentrations (Figure S3). CXCL9 concentrations in the VS YWH were comparable with those of the NI group, but significantly lower than those of the VNS group. The concentrations of iFABP were significantly higher in VS compared to NI, consistent with the predictions from the random forest classification model. LBP, sCD14 and CXCL10 concentrations were significantly elevated in VS and VNS compared to NI, while CCL5 concentrations were not significantly different among the three groups.

### 3.2. Differential Gene Expression Profile in VS Compared with VNS and NI

**Figure 4:**
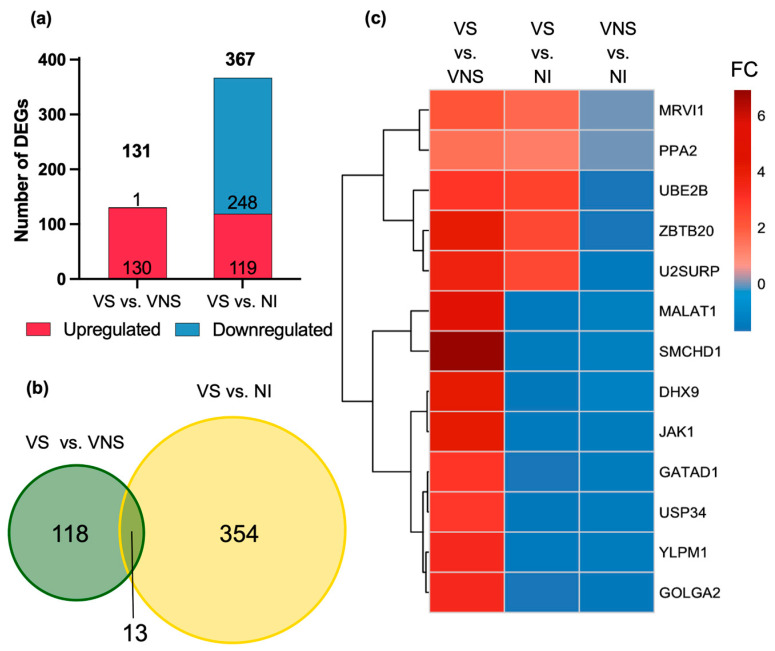
Differential expression of genes (DEGs) in VS compared with VNS and NI. (**a**) DEG analysis was performed to compare VS with VNS and NI. Differentially expressed genes (DEGs) showing an absolute FC ≥ 1.3 and FDR ≤ 0.05 were considered significantly altered. Red: upregulated DEGs; blue: downregulated DEGs. (**b**) DEGs from the two comparisons were plotted as a Venn diagram to study the gene overlap between the two VS comparisons. (**c**) Fold change (FC) of 13 DEGs shared across all the comparison was plotted as a heatmap and clustered based on FC.

To enhance the depth of profiling and better characterize the viral suppression profile, an analysis of the peripheral blood cell transcriptome was conducted, encompassing over 20,000 genes per sample. In the comparison between the VS and VNS groups, a distinct gene expression profile emerged, with 130 upregulated DEGs and only 1 downregulated DEG. In contrast, the comparison between VS and NI identified 367 DEGs, with about one-third (n = 119) upregulated and two-thirds (n = 248) downregulated (Figure 4a). The comparison between VNS and NI revealed more than 1000 DEGs, including 227 upregulated and 776 downregulated, highlighting the significant impact of viral replication on the peripheral blood cell transcriptome, consistent with our previous finding [19] (Figure S4a).

The VS group demonstrated a distinct gene expression profile compared to both the VNS and NI groups. Of the total 131 DEGs identified between VS and VNS, 118 DEGs were unique to this comparison. Similarly, the comparison between VS and NI identified 367 DEGs, of which 354 DEGs were unique. Only 13 DEGs were shared across the two VS comparisons, highlighting the unique transcriptional profile associated with viral suppression (Figure 4b).

The 13 shared DEGs displayed notable patterns in expression across the groups (Figure 4c). Five DEGs (*MRVI1*, *PPA2*, *UBE2B*, *ZBTB20*, and *U2SURP*) were consistently upregulated in VS compared with VNS and NI, suggesting their specificity to the viral suppression profile. In contrast, eight DEGs showed differential regulation: upregulated in VS compared with VNS but downregulated in VS compared with NI. Additionally, 11 of 13 shared DEGs overlapped with those identified in the VNS versus NI comparison, and all 11 DEGs were downregulated in VNS compared with NI, except *MRVI1* and *PPA2*, which were not differentially expressed.

### 3.3. Unique Canonical Pathway Perturbation and Gene Expression Patterns Associated with Viral Suppression

**Figure 5:**
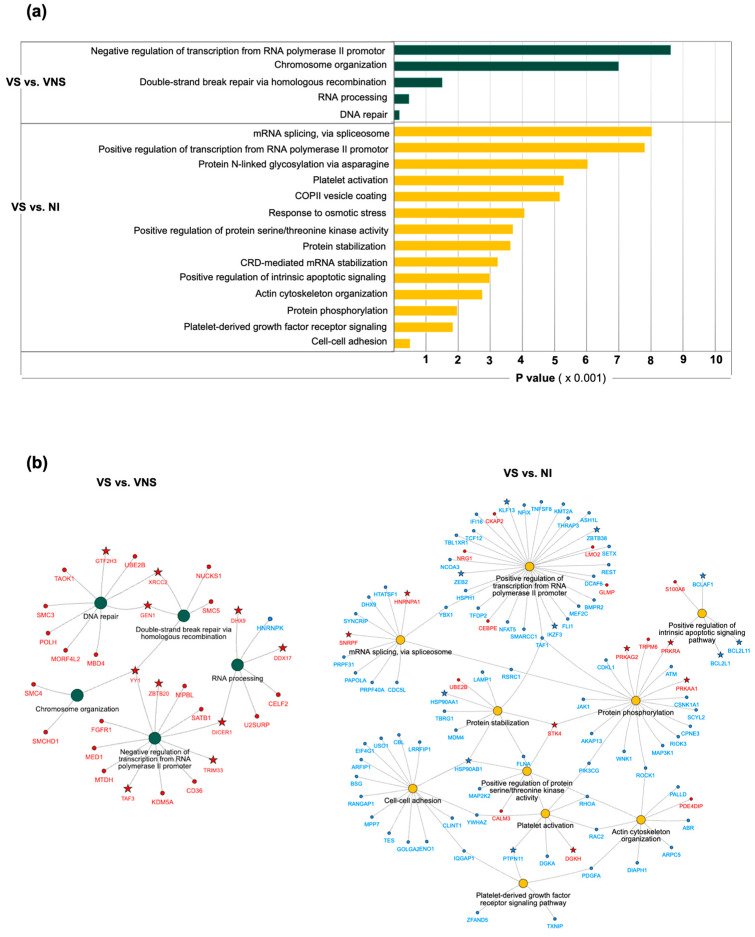
Functional enrichment and network analysis. (**a**) Functional enrichment analysis was performed to characterize the DEGs in VS compared with VNS or NI using a *p* value cut off ≤ 0.001. Green bars: Pathways perturbed by DEGs when VS compared with VNS; yellow bars: pathways perturbed by DEGs when VS compared with NI. (**b**) Network analysis of significant pathways shows DEGs connecting the pathways for VS compared with VNS (left panel) or VS compared with NI (right panel). Nodes: Pathways; edges: DEGs connecting the pathways. Green circles: VS compared with VNS; yellow circles: VS compared with NI; red: upregulated DEGs; blue: downregulated DEGs within each pathway; Star: candidate DEGs associated with viral suppression in both VS comparisons.

DEGs from the comparison of VS with VNS groups showed five perturbed pathways mainly involved in DNA repair, double-strand break repair via homologous recombination, chromosomal organization, RNA processing, and negative regulation of transcription by RNA polymerase II. DEGs from VS compared with NI showed enrichment of fourteen canonical pathways associated with positive regulation of intrinsic apoptotic signaling, positive regulation of transcription by RNA polymerase II, mRNA splicing, protein stabilization, phosphorylation, regulation of serine/threonine kinase activity, cell–cell adhesion and platelet activation, among others (Figure 5a).

To identify the candidate genes associated with viral suppression, network analysis was conducted on the perturbed pathways. Genes from the same family expressed in the same direction within a single pathway or across multiple pathways were selected as candidate genes, reflecting a pattern of co-expression associated with viral suppression (Figure 5b). In VS compared with VNS, the *XRCC2/RAD51* and *Gen1/RAD2* genes were upregulated in DNA repair and double-strand break repair pathways. Additionally, the RNA processing pathway showed upregulation of multiple genes encoding for RNA regulating enzymes, including helicases such as *DDX17*, *DHX9*, and miRNA-processing enzyme *Dicer1*. Multiple transcription factors were upregulated in canonical pathways related to DNA or double-strand nucleic acid break repair, negative regulation of transcription by RNA polymerase II, and chromosome organization (*GTF2H3*, *TAF3* and *YY1*, a member of the zinc finger protein family, were dysregulated across multiple pathways). Moreover, in the negative regulation of transcription by RNA polymerase II pathways, transcriptional repressors like *TRIM33* and *ZBTB20* were upregulated.

In VS compared with NI, pathways such as positive regulation of intrinsic apoptotic signaling showed downregulation of multiple genes (*BCL2L11*, *BCL2L1* and *BCLAF1*) belonging to the BCL2 family (Figure 5b). Similarly, the positive regulation of the RNA polymerase II pathway demonstrated downregulation of several transcription repressors such as *KLF3*, and *ZEB2*, as well as with transcription factors *IKZF3* and *ZBTB38*, all belonging to the zinc finger protein family. Meanwhile, genes like *HSP90AB1* and *HSP90AA1* from the heat shock protein 90 family were downregulated across multiple pathways such as protein stabilization, positive regulation of protein serine/threonine kinase activity, and cell–cell adhesion. Both platelet-derived growth factor receptor signaling and the platelet activation pathway displayed downregulation of tyrosine phosphatase *PTPN11/SHP2* gene. In contrast, upregulation of *SNRPF* and *HNRNPA1* genes was observed in mRNA splicing via spliceosome pathway. Additionally, cellular kinases such as *STK4*, *PRKRA*, *PRKAG2*, *PRKAA1*, and *DGKH* were upregulated and perturbed across multiple pathways. Upregulation was profound in the genes perturbed in the pathways by VS compared with VNS, while a prominent downregulation of gene was seen in VS compared with NI.

DEGs from VNS compared with NI mapped to over 30 canonical pathways, highlighting the significant impact of viral replication. These pathways were mainly related to immune/interferon response, defense responses to viruses, viral processes and transcription regulation, among others (Figure S4b,c).

**Figure 6:**
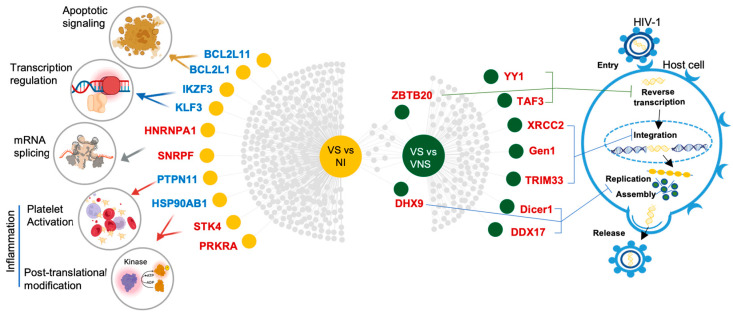
Candidate DEGs/genes associated with viral suppression. Genes playing critical roles in regulating molecular mechanisms that contribute to the HIV-1 control at different levels were identified in VS compared with VNS. Genes regulating apoptosis, transcription, pathways involved in post-translation modification (PTM) (such as protein stabilization, phosphorylation, regulation of serine/threonine kinase activity), as well as inflammation (platelet activation) were identified in VS compared with NI. Symbols: Dots—DEGs; grey dots—total DEGs in both VS comparisons; green dots—candidate DEGs identified in VS vs. VNS; yellow dots—candidate DEGs identified in VS vs. NI; candidate DEGs labeled by gene symbol—red: upregulated; blue: downregulated.

Candidate DEGs associated with viral suppression were further identified based on the differential gene expression and pathway analysis as potential therapeutic targets for profiling sustained viral suppression (Figure 6). In the comparison of VS with VNS, candidate DEGs/genes that play critical roles in regulating molecular mechanisms such as DNA repair, RNA processing and transcriptional repression, which contributes to HIV-1 control at different levels, were identified. Genes regulating apoptosis, transcription, and pathways involved in post-translation modification (PTM) (such as protein stabilization, phosphorylation, regulation of serine/threonine kinase activity), as well as inflammation (platelet activation), were identified as candidate DEGs in the comparison of VS compared with NI.

To explore the relationship between plasma biomarker trends and corresponding transcript levels, the RNA expression levels (log_2_-transformed values) of genes encoding highly significant plasma biomarkers were examined (Figure S5). Consistent with the plasma biomarker concentrations, the log_2_ expression levels of *CXCL9* were significantly lower in the VS group compared to the VNS group. *CXCL10* levels also showed significant differences between the VS and VNS groups; however, the VS levels did not differ significantly from NI. The expression levels *CCL5*, *CD14*, *LBP*, *FABP2* and *CXCL8* (encoding IL-8) did not differ significantly across the study groups.

## 4. Discussion

This study employs a novel, multi-omics approach, analyzing plasma biomarkers and peripheral blood cell gene expression to profile host responses in youth with behaviorally acquired HIV infection and ART-suppressed viral replication. The YWH participants were principally African American males, a population at heightened risk for HIV infection, who were treatment-naïve before enrollment, early in their disease course with CD4 T cell counts within the normal range and as a group have fewer comorbidities than adults [6,45]. Comparison between virally suppressed and non-suppressed YWH is enhanced due to similarity in their CD4 T cell counts, demographics, treatment regimens, and disease stages, while the inclusion of a sex- and age-balanced uninfected youth cohort provided a robust comparative group.

The plasma biomarkers analyses identified unique viral suppression profiles that distinguish virally suppressed youth from virally non-suppressed youth and those with no infection. The nuanced computational approach highlighted a subset of biomarkers predictive of a VS profile, revealing whether higher or lower concentrations of each biomarker are more indicative and offering insights into their directional impact on viral suppression. The strong AUC-ROC values of both VS models demonstrate their robust discriminatory power, validating the biomarker panel’s effectiveness in distinguishing VS from both VNS and NI.

Compared with VS youth, youth with detectable viral replication display higher levels of biomarkers across multiple immune pathways associated with ongoing inflammation through interferon inducible chemokines (CXCL9 and CXCL10), gut intestinal barrier dysfunction (iFABP), and immune activation (CCL5 and IL-2Ra) [8,9,20,46]. When compared to NI, VS youth continue to display higher concentrations of some biomarkers of inflammation, macrophage activation, persistent gastrointestinal mucosal permeability, and vascular injury. However, VS youth display normalization of several biomarkers associated with increased morbidity, such as CXCL9, IL-6, sCD163, and TNFα, that remain persistently elevated in suppressed older adults [47,48,49]. These findings suggest that viral suppression, when achieved early in the disease, may normalize inflammatory pathways commonly associated with HIV-related comorbidities. [8,50,51].

While insightful, plasma biomarker analysis is limited by factors such as the range of detection, a high degree of variability, and the possibility for overlooking novel biomarkers. However, the inclusion of gene expression analysis of peripheral blood cells can expand the breadth of discovery.

Candidate DEGs/genes from canonical pathways perturbed in VS compared with VNS youth reveal molecular mechanisms contributing to HIV control, including upregulated DNA damage response (DDR) pathways, such as DNA repair and double-strand break repair via homologous recombination, both essential for genomic integrity and cellular function, as well as RNA processing and transcription regulation pathways. Although HIV-1 impairs DDR [52,53], the upregulation of DNA repair genes suggests enhanced repair processes that promote cellular homeostasis and improved viral control. For instance, *RAD51* restricts viral integration by enhancing DNA repair [54]. RNA helicases involved in RNA processing regulate viral replication, possibly limiting the emergence of escape variants by targeting viral RNA and enhancing immune responses [55,56,57]. Elevated expression of RNA helicases in VS compared to VNS highlights the important role of RNA metabolism in sustaining viral suppression.

Upregulation of transcriptional regulators, including genes encoding zinc finger proteins *YY1* and *ZBTB20*, was observed in VS compared with VNS. *YY1* represses HIV-1 transcription by recruiting corepressor complexes and promoting chromatin remodeling at the LTR [58,59]. *ZBTB20*, linked to HIV-1 resistance in highly exposed seronegative HIV-1-resistant sex workers [38], was upregulated in VS and was among the 13 shared DEGs, suggesting a protective role in preventing viral transcription. Additionally, *TRIM33* restrains HIV-1 infection by targeting viral integrase [60], while *TAF3*, a transcription cofactor, contributes to promotor recognition and selectivity [61].

Upregulation of DEGs involved in DDR, RNA processing, and transcriptional repression pathways suggests that viral control by ART is optimized in individuals with these transcriptomic profiles. These pathways collectively enhance genomic stability, suppress viral replication, and strengthen cellular defenses.

In VS, compared with NI, perturbed pathways encompass diverse biological processes, including the regulation of apoptosis, transcription, pathways involved in post-translation modification (PTM) (such as protein stabilization, phosphorylation, regulation of serine/threonine kinase activity), and inflammation (platelet activation), among others. Downregulation of *BCL2* genes, involved in apoptosis regulation, suggests a role in viral reservoir maintenance and immune evasion. *BCL2* inhibitors, shown to have anti-HIV effects in pre-clinical studies, could target latent reservoirs and promote infected cell clearance [62,63]. Perturbations in zinc-finger genes (*IKZF3*, *ZBTB38*, *KLF3*), involved in RNA polymerase II regulation, point to altered transcriptional control of HIV replication and persistence [64,65,66,67]. Upregulation of mRNA splicing genes, such as *HNRNPA1*, suggests that modulating splicing mechanisms could lead to viral control. *HNRNPA1* overexpression reduces HIV-1 replication by affecting the localization of viral mRNA [68]. Targeting splicing pathways might provide an effective strategy to limit viral persistence [69,70,71].

*PTPN11/SHP2* and heat shock protein 90 (*HSP90*), which play roles in regulating cellular signaling, cell survival and immune response, are downregulated in VS compared with NI. Inhibition of *HSP90* prevents HIV-1 reactivation in CD4+ T cells by suppressing the NF-κB pathway, a crucial regulator of HIV-1 latency and reactivation [72,73]. Upregulation of cellular kinases indicates alterations in PTM pathways, which regulate key cellular processes. Investigating these PTM processes may offer valuable insights into targeting the proteasomal regulation of both viral and host proteins, potentially restricting HIV-1 replication and limiting the survival of infected cells [74].

Targeting candidate genes involved in apoptosis regulation, transcriptional control, splicing mechanisms, cellular signaling, immune regulation, and post-translational modification (PTM) pathways could provide new therapeutic strategies to limit viral persistence and enhance HIV treatment outcomes in virally suppressed individuals. Transcriptome studies in older adults on treatment revealed perturbations in transcriptional control and immune regulation, underscoring the relevance of these pathways in HIV persistence and progression [14,15]. The findings in YWH align with these studies, but also reveal unique pathways related to PTM, highlighting developmental differences that may influence HIV pathogenesis and treatment responses in younger populations.

Additionally, both suppressed and non-suppressed YWH showed comparable viral loads and CD4 T cell counts at entry, with the VS group even starting with higher viral loads, yet suppression outcomes at the end of study varied. Lymphocyte subpopulations including CD4 T cells, CD8 T cells, and CD19 B cells were similar between VS and VNS YWH at the end of study, indicating similar cellular composition across the groups that did not directly influence viral suppression outcomes. This suggests that in YWH, the outcome of viral suppression is largely independent of clinical pretreatment markers, such as viral setpoint or baseline CD4 T cell counts [75]. Some non-suppressed youth had intermittent low but detectable viral levels, indicating that suppression status could change over time. These fluctuations in viral levels, even at low concentrations, underline the need to consider dynamic changes in viral load over time.

The ART regimen used in this study was consistent with the standard of care at the time and was consistent across YWH groups to mitigate the possible impact of ART on gene expression profiles. An omics study investigating pre-exposure prophylaxis (PrEP) effects in HIV-negative individuals revealed minimal global changes in host gene expression, with no DEGs identified in the blood [76]. Achieving and maintaining viral suppression below 50 copies/mL of HIV-1 RNA significantly reduces sexual transmission risk, a critical goal in public health [77]. However, sustained suppression is still associated with challenges like chronic immune activation, metabolic dysregulation, and vascular inflammation, underscoring the importance of identifying specific markers to better understand and classify suppression profiles.

Despite the limited overview of expressed genes in PBC with the microarray analysis, the results identified multiple markers and candidate genes, delineating a distinct bioprofile for virally suppressed YWH compared with non-suppressed YWH or youth with no infection. However, further validation with a larger sample size is warranted to enhance the precision of computational methodologies and the robustness of the findings.

RNA expression levels (log_2_-transformed values) of genes encoding highly significant plasma biomarkers showed *CXCL9* expression aligned with CXCL9 plasma concentrations, indicating transcriptional regulation, likely driven by IFNγ mediated immune activation, suggesting minimal post-transcriptional modulation [78]. In contrast, *CXCL10* RNA levels do not mirror plasma trends, likely due to regulation by multiple factors, including type I interferons and TNFα, which affect plasma protein independently of transcription [78,79]. Similarly, *CD14* and *LBP* transcript levels are discordant from plasma levels, likely reflecting post-transcriptional processes such as protein shedding or cleavage from cell surfaces contributing to plasma concentrations [80]. Correlating RNA expression levels with plasma biomarker concentrations is complex, potentially due to the influence of transcriptional and post-transcriptional modifications, including factors such as mRNA stability, translation efficiency, protein turnover, and proteolytic shedding, which can impact plasma biomarker levels independently of RNA expression.

Individual bioprofiles highlight key factors influencing sustained viral suppression. Elevated inflammatory markers, associated with immune activation, gut dysfunction, and vascular injury, suggest that persistent inflammation may impede effective viral suppression on ART in non-suppressed youth. In contrast, early viral suppression may help normalize inflammatory pathways and key biomarkers linked to HIV-related comorbidities. Upregulated genes in canonical pathways associated with cellular homeostasis, including DNA repair, RNA processing, and transcription regulation, could play a protective role by preventing viral breakthrough. Bioprofiling may also aid in developing expression-based diagnostic classifiers for early detection of resurgent virus [81,82], and improve the identification of individuals at risk for viral rebound. This approach not only advances patient management but also enhances our understanding of the immunological and molecular dynamics underlying HIV infection. Ultimately, leveraging bioprofiles in clinical practice will enable more personalized treatment strategies, improving health outcomes for individuals living with HIV.

## 5. Conclusions

This study used a multi-omics approach to evaluate plasma biomarkers and gene expression profiles in YWH on ART, providing valuable insights into viral suppression. It identified a unique viral suppression profile in YWH, distinct from both non-suppressed YWH and uninfected youth. Perturbed canonical pathways, along with candidate markers and genes, that may optimize ART-mediated viral suppression and present potential therapeutic targets to limit viral persistence and improve HIV treatment outcomes were identified. The findings also highlight the importance of bioprofiling.

## Figures and Tables

**Table 1:** Demographic and clinical profiles of study groups (N = 52).

Characteristics		No Infection (NI)	Infection	
			VS (VL ≤ 50)	VNS (VL > 50)
**Sample Size (N)**		25	19	8
**Age (years) ^a^**		22 [20, 23]	24 [24, 26]	25 [23, 25]
**Male (%)**		68	84	75
**African American (%)**		80	79	88
**ART Regime ^b^ (%)**	**NRTI**	NA	100	95
	**NNRTI**	NA	25	37
	**PI**	NA	88	69
**Days on ART ^a^**		NA	1043 [921, 1061]	959 [849, 1024]
**HIV-1 RNA plasma at entry (copies/mL) ^a^**		NA	22,607 [11,920, 42,355]	16,504 [5645, 20,469]
**HIV-1 RNA plasma at end of study (copies/mL) ^a^**		NA	≤50	6706 [134, 18,152]
**CD4 T cells at entry** **(number/μL) ^a^**		NA	442 [325, 490]	345 [282, 622]
**CD4 T cells at end of study (number/μL) ^a^**		751 [463, 859]	760 [611, 890]	548 [369, 702]
**CD4 T cells nadir** **(number/μL) ^a^**		-	451 [374, 470]	394 [311, 527]

^a^ Median [25th, 75th quartile]. ^b^ Percentage of youth receiving respective classes of ART. NA: not applicable. NRTI: nucleoside reverse transcriptase inhibitors. NNRTI: non-nucleoside reverse transcriptase inhibitors. PI: protease inhibitors. VL: viral load. Statistical significance defined as *p* value < 0.05.

## Data Availability

The data were uploaded to dbGaP [83] and will be released upon the publication of this manuscript (Accession ID phs002090.v1.p1).

## Supplementary Materials

The following supporting information can be downloaded at: https://www.mdpi.com/article/10.3390/cells14040285/s1, Table S1: End of study percentages of lymphocyte subpopulation, Figure S1: AUC-ROC curve; Figure S2: Beeswarm plot of RF model of VNS compared with NI; Figure S3. The top biomarker distinguishing VS from VNS and NI; Figure S4: a. DEG analysis for VNS vs. NI, b. functional enrichment for VNS vs. NI, c. network analysis of VNS vs. NI; Figure S5: RNA expression levels (log2-transformed values) of genes encoding highly significant plasma biomarkers.
