# Supplementary material for: Youth Who Control HIV on Antiretroviral Therapy Display Unique Plasma Biomarkers and Cellular Transcriptome Profiles Including DNA Repair and RNA Processing
Source: Cells. 2025 Feb 15;14(4):285. doi: 10.3390/cells14040285 (PMC11853983; doi:10.3390/cells14040285)
Supplement: Supplementary file 1 [file cells-14-00285-s001.zip › cells-3411068-supplementary.pdf]

Table S1. End of study percentages of lymphocyte subpopulations			
Percent of cells*	No Infection (NI)	Infection	
		VS (VL ≤ 50)	VNS (VL > 50)
**Total CD4 T cells[a]**	**46.1 [40.8, 50.4]**	**37.5 [30.3, 40.8]**	**31.2 [25.3, 38.0]**
Naïve CD4 T cells[a]	41.4 [37.7, 59.4]	41.0 [39.1, 45.8]	38.1 [34.7, 41.2]
Effector Memory CD4 T cells[a]	19.5 [14.1, 24.7]	28.6 [21.1, 36.4]	20.5 [14.0, 27.0]
**Total CD8 T cells[a]**	**24.5 [20.6, 27.5]**	**36.7 [32.2, 40.2]**	**40.8 [37.0, 44.9]**
**Total CD19 B cells[a]**	**13.6 [11.2, 16.1]**	**8.3 [6.7, 10.6]**	**7.1 [3.9, 7.3]**

* Median [25th, 75th quartile].

[a] Total Percentage of CD4,CD8 and CD19 lymphocyte subsets based on CD45. Percent CD4 naïve (CD45RA+ and CCR7+) and effector memory ($CD45RA^-$ and $CCR7^-$) are shown as a percent of total CD4 T cells.

Percentages of lymphocytes subpopulation were comparable between VS and VNS YWH groups based on one-way Anova (P < 0.05) whereas NI was significantly different from VS and VNS for total CD4 T cells, CD8 T cells and CD19 B cells.

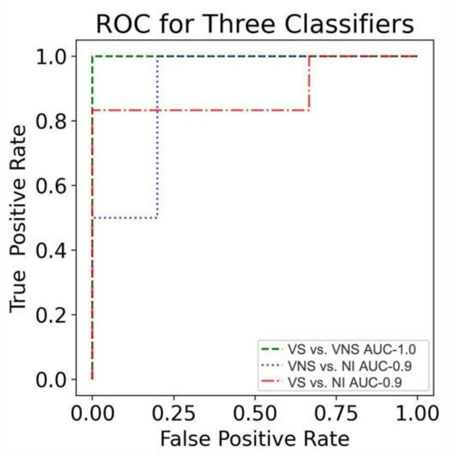

Figure S1 AUC-ROC curve. AUC of VS vs. VNS; VS vs. NI and VNS vs. NI for RF model with 70-30% training and test split where for each classification model, 70% of the data was randomly used to train the model, while 30% unseen data was used to test.

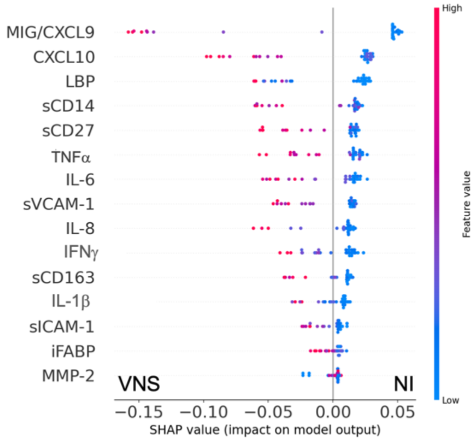

Figure S2. Beeswarm plot of RF model showing the top fifteen biomarkers that distinguish VNS compared with NI

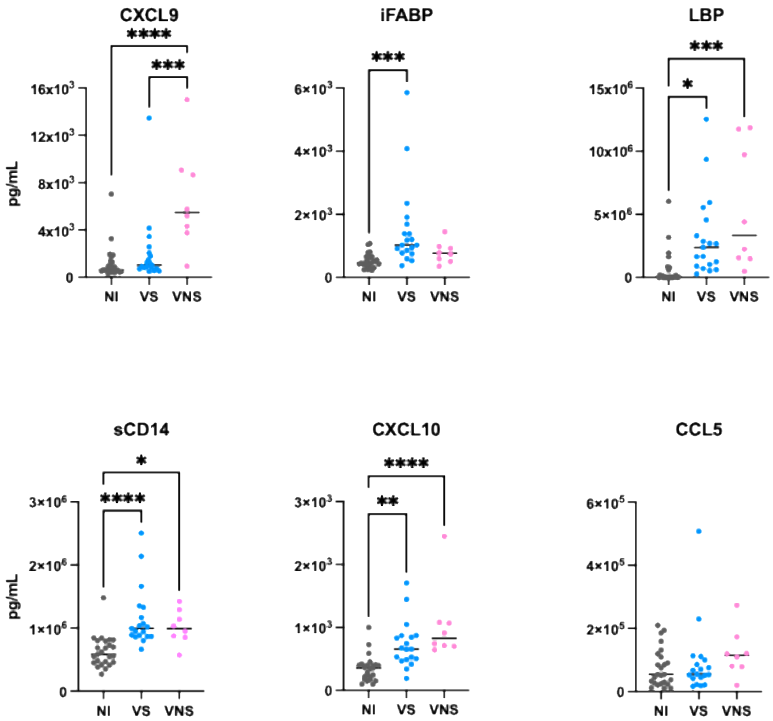

Figure S3. The top biomarker distinguishing VS from VNS and NI. The top biomarker from RF analysis was further examined to assess the significance of the differences between the groups. One-way ANOVA was applied with significant p value < 0.05; median biomarker values was shown. Symbols: dots, participants

Grey dots: NI; Blue dots: VS; Pink dots: VNS

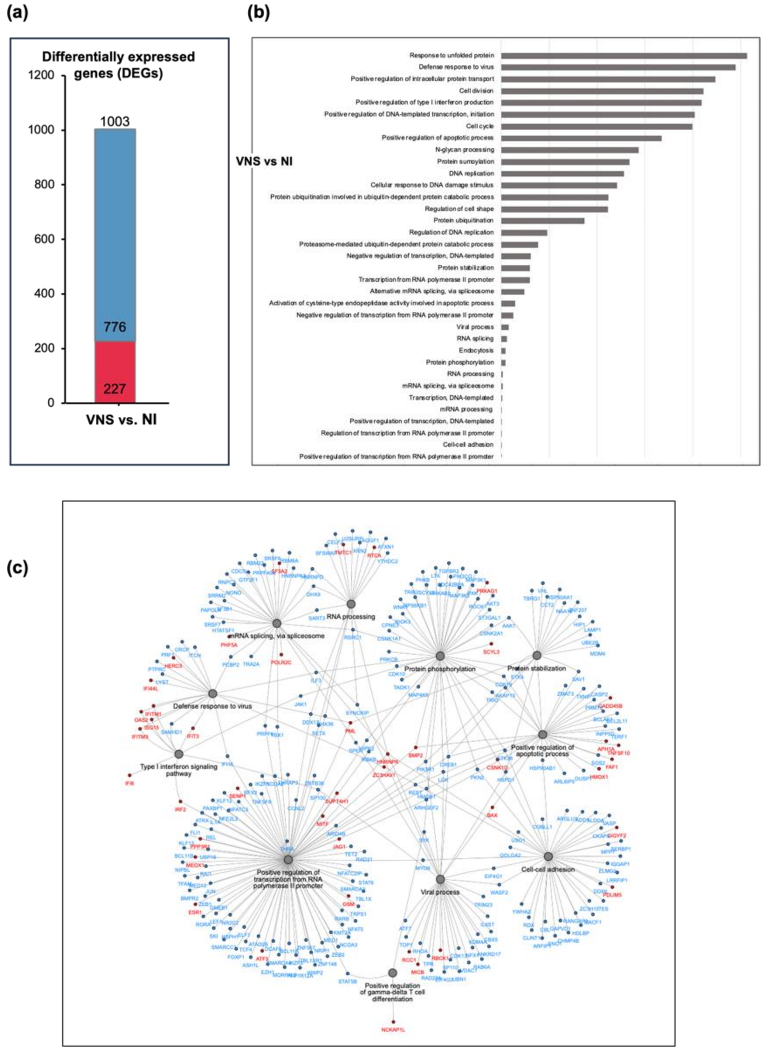

Figure S4. (a) Differential gene expression analysis was performed to compare VNS with NI. Differentially expressed genes (DEGs) showing an |FC| ≥ 1.3 and FDR ≤ 0.05 were considered significantly altered. Red: Upregulated DEGs; Blue: downregulated DEGs.
(b) Functional Enrichment and network analysis. Functional enrichment analysis was performed to characterize the DEGs in VNS compared with NI using P value cut off ≤ 0.001.
Grey bars: Pathways perturbed by DEGs in VNS compared with NI.
(c) Network analysis of significant pathways shows DEGs connecting the pathways for VNS compared with NI. Nodes: Pathways; edges: DEGs connecting the pathways; Red: Upregulated DEGs; Blue: downregulated DEGs within each pathway

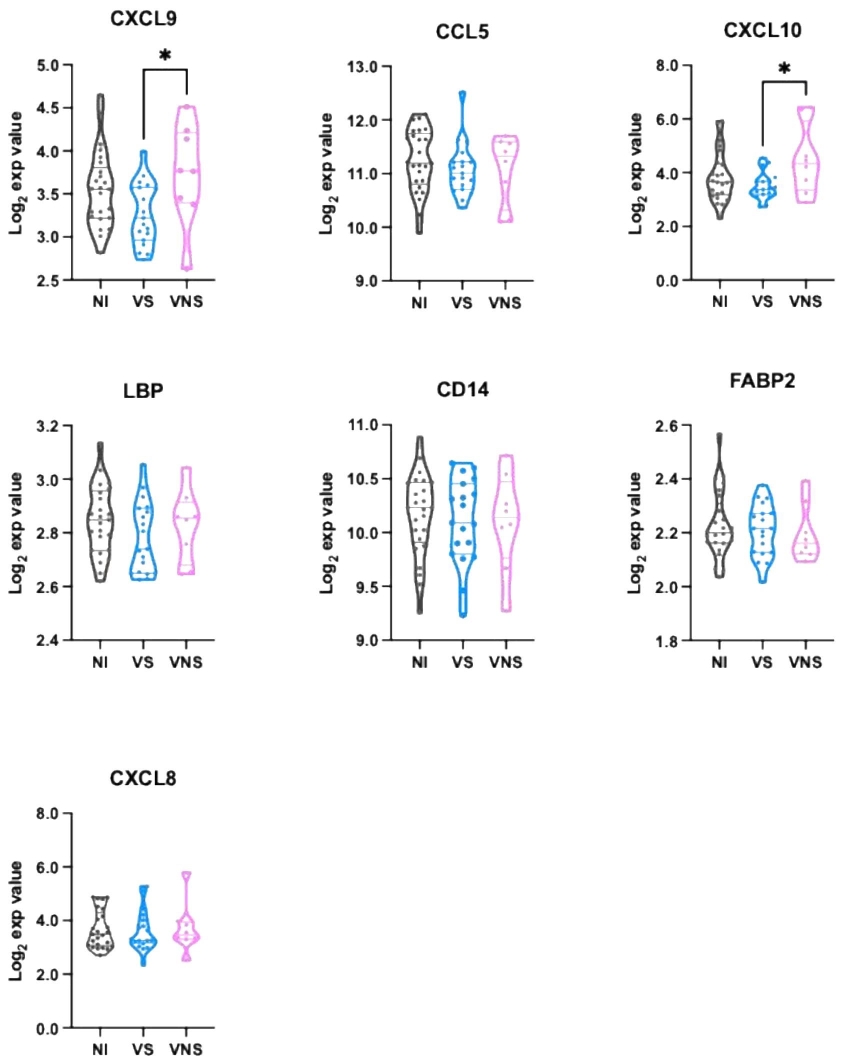

Figure S5. RNA expression levels (log$_2$-transformed values) of genes encoding highly significant plasma biomarkers were visualized using Violin plots. The distribution of RNA expression levels for each selected gene across study groups was assessed using GraphPad Prism 10.0.3 (GraphPad Software, San Diego, CA, USA). Statistical significance of differences between groups was assessed using One-way ANOVA ($p < 0.05$).

Symbols: circles, participants

Grey: NI; Blue: VS; Pink: VNS
